# Skin Cancer Detection Using Infrared Thermography: Measurement Setup, Procedure and Equipment

**DOI:** 10.3390/s22093327

**Published:** 2022-04-26

**Authors:** Jan Verstockt, Simon Verspeek, Filip Thiessen, Wiebren A. Tjalma, Lieve Brochez, Gunther Steenackers

**Affiliations:** 1 InViLab Research Group, Department Electromechanics, Faculty of Applied Engineering, University of Antwerp, Groenenborgerlaan 171, B-2020 Antwerpen, Belgium; simon.verspeek@uantwerpen.be (S.V.); gunther.steenackers@uantwerpen.be (G.S.); 2 Department of Plastic, Reconstructive and Aesthetic Surgery, Multidisciplinary Breast Clinic, Antwerp University Hospital, University of Antwerp, Wilrijkstraat 10, B-2650 Antwerp, Belgium; filip.thiessen@clinic12b.be; 3 Gynaecological Oncology Unit, Department of Obstetrics and Gynaecology, Multidisciplinary Breast Clinic, Antwerp University Hospital, University of Antwerp, Wilrijkstraat 10, B-2650 Antwerp, Belgium; wiebren.tjalma@uza.be; 4 Department of Dermatology, Ghent University Hospital, C. Heymanslaan 10, B-9000 Ghent, Belgium; lieve.brochez@ugent.be

**Keywords:** infrared thermography, skin cancer, cooling setup, infrared camera, skin lesion, measurement setup

## Abstract

Infrared thermography technology has improved dramatically in recent years and is gaining renewed interest in the medical community for applications in skin tissue identification applications. However, there is still a need for an optimized measurement setup and protocol to obtain the most appropriate images for decision making and further processing. Nowadays, various cooling methods, measurement setups and cameras are used, but a general optimized cooling and measurement protocol has not been defined yet. In this literature review, an overview of different measurement setups, thermal excitation techniques and infrared camera equipment is given. It is possible to improve thermal images of skin lesions by choosing an appropriate cooling method, infrared camera and optimized measurement setup.

## 1. Introduction

In 2020, more than 19 million new cases of cancer were diagnosed, with an estimation of around 10 million deaths. Non-melanoma of the skin accounts for 6.2% of the total amount of new cases, while melanoma of the skin accounts for 1.7% of new cases [1,2]. Melanoma incidence rates have tripled in Europe over the past four decades, similar to the United States. According to the World Health Organization, the total annual economic cost of cancer is estimated to be over 1 trillion euros. These figures are expected to rise further due to increasing population and life expectancy [1]. Cancers of the skin are the most common type of cancer in humans. “Skin cancer” refers to a number of pathological entities originating from various cells of the epidermis and dermis. Skin cancer is mainly divided into melanoma and non-melanoma skin cancer is further divided in Merkel cell cancer and keratinocyte cancers such as basal cell carcinoma and squamous cell carcinoma [2]. Early detection of the cancer increases survival rates and leads to less expensive, more effective treatments that often have less impact on patients’ quality of life [3]. Regular screening is recommended for populations at moderate to high-risk, such as annual breast cancer screening for older women or annual lung cancer screening for those between 55 and 74 years of age with a smoking history [4,5].

### 1.1. Today’s Screening Methodology

Skin cancer screening nowadays includes a total body skin examination (TBSE) [4]. This is a relatively quick, inexpensive and noninvasive procedure in which dermatologists perform skin examinations to differentiate possible malignant and benign skin lesions. Skin classification depends on proper training and the experience of the dermatologists [6].

Breslow thickness, i.e., the penetration of malignant melanocytes in the skin measured in millimeters, is the most important prognostic factor in melanoma patients [7]. Currently, the diagnosis of melanoma is made by naked-eye and dermoscopic examination using the ABCDE classification by Nachbar and Stolz [8], which stands for Asymmetry, Border irregularities, Colour differences, large Diameter, Evolution over time. Even when performed by skilled dermatologists the technique has a relatively low specificity (56–65%) and moderate sensitivity (47–89%) [9]. Introduction of dermoscopy to trained dermatologists increased sensitivity from 69% to 87% and specificity from 88% to 91% [10]. In general, dermoscopy with polarized light is used as an additional technique to highlight additional features in skin lesions [11]. However, to exclude false-negative findings that could lead to metastasis and death, excisions are performed. These excisions are invasive and often unnecessary, as melanoma is detected for every 10 to 60 biopsies performed [9,12].

In this review, the focus is on studies that have been performed using various measurement setups, procedures and equipment for infrared thermography to diagnose skin cancer in humans. Some basic findings and concepts of thermography are also presented. Post-processing of IR images, processing algorithms and classification are outside the scope of this review. The purpose of this review is to summarize recent developments and new perspectives for future research on skin cancer diagnosis with infrared thermography.

### 1.2. Biomedical Infrared Thermography

Infrared thermal imaging is rarely used in biomedical applications, although there were early proponents and supporters of this new technology. The root cause is probably the unsatisfactory results of passive thermography and the use of bulky first-generation IR cameras and their limited performance [13,14,15]. Infrared thermography (IRT) has been continuously improved in recent years and is now widely used in scientific research and industry [15,16]. In the last decade, more and more researchers have rediscovered their interest in biomedical applications based on the well-described fundamentals of infrared thermography [13,17,18]. IRT is used in medical science for example, to detect breast cancer, psoriasis, for fever screening, in dentistry, or even in breast reconstruction with DIEP flaps [19,20].

**Figure 1:**
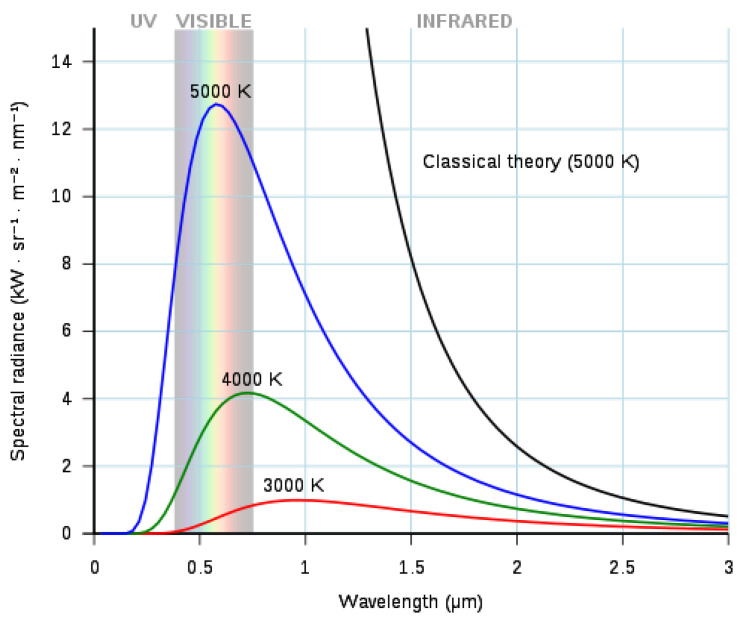
Radiance of blackbodies for various temperatures.

Infrared radiation or thermal radiation, which is part of the electromagnetic spectrum, is emitted by any object above the absolute zero temperature (−273.15 °C) [13,21]. Infrared radiation is temperature dependent [22]. The properties of this radiation are described by Planck’s law, which states that the spectral radiance $M_{\lambda }$ of a perfect emitter (blackbody) is given by Equation (1) and Figure 1 [23].

(1) $$M_{\lambda }\left(\lambda ,T\right)=\frac{2hc{}^{2}}{\lambda^{5}}\frac{1}{e^{\frac{hc}{\lambda kT}}-1}$$

where λ is the wavelength, *T* is the absolute temperature, *h* is the Planck constant, *k* is the Boltzmann constant and *c* is the speed of light in a vacuum.

According to Vollmer [24], emissivity ϵ is a measure of the efficiency in which an object radiates thermal energy and is characterized by the percentage of thermal energy (radiation) from the material’s surface being emitted relative to that radiated by a perfect emitter (a black body) at the same wavelength and temperature. Emissivity is a dimensionless number between 0 and 1. An emissivity value of 1 corresponds with a black body being a perfect emitter of heat energy and a perfect thermal mirror corresponds with an emissivity value of 1. As reported by Lee and Minkina, the commonly accepted emissivity ϵ of human skin, independent of the skin pigmentation, is 0.98±0.01 for λ>2μm, which makes human skin a close to perfect black body [15,25,26,27]. Many studies in the past have confirmed the emissivity of 0.98±0.01 [28,29,30,31,32,33]. In 2009, Sanchez-Marin et al. proposed a new approach to evaluate the emissivity of human skin [34]. They concluded that human skin follows Lambert’s law of diffuse reflectance and that the emissivity of skin is almost constant between 8 and 14 μm [34]. Charlton et al. conducted a study on the influence of constitutive pigmentation on the measured emissivity of human skin [35]. They collected data from participants with varying pigmentation according to the Fitzpatrick scale. Charlton et al. concluded that human skin emissivity is not affected by skin pigmentation [35]. They also advocate the use of an emissivity ϵ=0.98 for general use [35]. Several other parameters also affect the emissivity ϵ. Material, surface structure, angle of observation, wavelength, and temperature all have an effect on emissivity, with material being the most important parameter [23]. Bernard et al. showed that topical treatment of human skin with various substances such as ultrasound gel, disinfection, ointment, etc. has an effect on the emissivity of human skin [36]. They showed that it is necessary to integrate emissivity into the calculation of human skin temperature considering the environment and its temperature since the measured surface temperature is a function of emissivity [36]. Spurious radiation originating from the environment and reflected from the sample can be ignored because human skin is a nearly perfect blackbody. Hardy and Muschenheim concluded that dead skin can be considered as a perfectly black surface with an emissivity of ϵ=1 [31].

**Figure 2:**
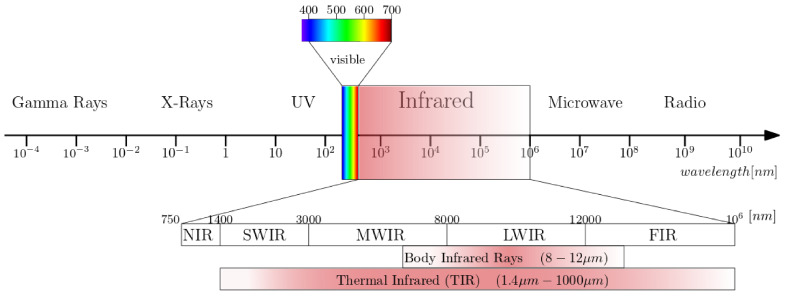
The electromagnetic spectrum with a subdivision for infrared wavelengths.

Figure 2 illustrates the electromagnetic spectrum and the IR spectral band in a finer scale. The boundaries between different IR spectral regions can vary. Infrared radiation has a wavelength range of 0.75 (0.78)–1000 μm. According to the ISO 20473 scheme the subdivision is as follows; Near-infrared or NIR (0.78–3 μm), Mid-infrared or MIR (3–50 μm) and Far-infrared or FIR (50–1000 μm). The boundaries that we adopt here are divided in smaller regions that divide up the band based on the response of various detectors [37]. The five sub-ranges are Near-infrared or NIR (0.7–1 μm), Short-Wave Infrared or SWIR (1–3 μm), Mid-Wave Infrared or MWIR (3–5 μm), Long-Wave Infrared or LWIR (7–14 μm) and Very-Long Wave Infrared or VLWIR (12–30 μm).

### 1.3. Skin Cancer and Infrared Thermography

When heat increases unexpectedly, it is an indication that something is wrong. For example, increased mechanical friction develops heat and causes wear, possibly leading to material failure [38]. Similarly, human heat is associated with many conditions such as inflammation and infection, and even in the time of Hippocrates, physicians used thermobiological diagnostics [39,40]. As a living organism, the human body attempts to maintain homeostasis, that is, an equilibrium of all systems within the body, for all physiological processes, which leads to dynamic changes in heat emission [41]. The result of a complicated combination of central and local regulatory systems is reflected in the surface temperature of an extremity. Core body temperature is maintained constant at depths larger than 20 mm [27]. Skin surface temperature is a useful indicator of health concerns or a physical dysfunction of near-to-skin processes [42].

Biomedical infrared thermography detects the emitted radiation on the human body surface and reveals the heterogeneous skin and superficial tissue temperature [43]. Infrared emissions from human skin at 27 °C are in the wavelength range of 2–20 μm, and peaks at 10 μm. Body infrared rays, a narrow wavelength range of 8–12 μm, is used for medical applications [22]. Another term commonly used in medical IR imaging is thermal infrared (TIR) [23]. This region includes the wavelengths 1.4–1000 μm, infrared emission is primarily heat or thermal radiation, hence the term thermography.

The use of infrared thermography for skin cancer is optimal because of the temperature changes and temperature distribution and it is noninvasiveness [42]. Skin cancer cells are enlarged compared to normal skin cells due to the high rate of uncontrolled cell division [40]. As a result of the high rate of cell division, cancer cells must convert more energy to run cellular processes [44]. This chemical process is called metabolism. Due to the high metabolism, there is a higher energy demand, which also leads to increased angiogenesis. Angiogenesis is the physiological process by which new blood vessels form from existing vessels to provide the necessary extra energy [45,46,47]. In conjunction with the increased energy requirements, melanoma skin lesions are thought to have a higher (Δ 2–4 K) temperature than the surrounding healthy skin [27,48,49,50]. Therefore, IR imaging for melanoma skin lesions is based on the detection of new blood vessels and chemical changes associated with a tumour development and growth [42,51]. Other skin tumor types, such as basal cell carcinoma may form an encapsulating layer of involved cells which acts like a thermal insulator, resulting in a delayed thermoregulatory process [27,52]. Similarly, González et al. [50] studied the vascularity of different skin lesions (melanoma and non-melanoma) and discovered that each cancer type has its own thermal signature.

## 2. Concepts of Thermography

### 2.1. Qualitative versus Quantitative Thermography

In qualitative thermography, the infrared data is presented as an image of the scene or sample and this thermogram is sufficient to analyze or interpret the problem. It finds its use in search and rescue operations where warm human bodies should be found, in site surveillance of places or in situations with poor visibility [53].

In contrast, quantitative thermography uses the ability to detect and record the temperature of each pixel. The infrared camera must be calibrated and take into account atmospheric interference, surface characteristics, emissivity, camera angle, distance etc. [54].

### 2.2. Passive versus Active Thermography in Biomedical Applications

Passive thermography investigates the sample in its steady state without application of an external thermal stress [13]. The passive approach tests materials and structures that are inherently at a different temperature than the ambient temperature [55]. Active thermography measures transient temperatures following an external thermal load. The thermal modulation can be cooling or heating of the sample. Thermal loading can be by conductive or convective heat transfer or by absorption of infrared radiation. The major drawback of thermography are the exogenous disturbances such as external heat sources, evaporative heat losses, etc., which can introduce biases or affect image quality [56].

#### 2.2.1. Passive Thermography

Passive thermography is the most commonly used thermal imaging technique [13]. Thermograms are taken from a sample in a steady state, so there is no external thermal excitation. The thermograms are examined for abnormal temperature differences, hot and cold spots or asymmetric temperature distributions, that indicate a potential health problem [42]. Passive thermal imaging is qualitative and the information that can be retrieved is rather limited [13]. Hot or cold spots on the skin surface are influenced by various subcutaneous factors such as metabolic processes or the presence of large blood vessels and bones, etc. Other external factors can also influence passive thermography measurements. The position of the patient, heat exchange conditions with the environment, recent ingestion of hot or cold beverages, time of the day, etc., all have an influence on the passive thermography measurements [43]. Other environmental conditions that may effect the measurements are: room temperature, relative humidity, air circulation flow and the intrinsic conditions of the examination room [57]. This results in limitations in the interpretation of thermograms. Interpretation of the diagnostic value of skin temperature distribution is difficult and requires careful preparation of the patient in stable environmental conditions [58]. To limit the influence of such factors, some authors [59] have attempted to devise rigorous measurement procedures, which unfortunately severely limit the feasibility of passive thermography in routine clinical practice [13].

#### 2.2.2. Active Thermography

Unlike passive thermography, active thermography requires thermal excitation, while an IR imaging device captures the dynamic temporal distribution of temperature [13]. Active thermography can be used to obtain quantitative information about the thermal properties of the sample. To use the quantitative data, a temperature calibration should be performed at known temperatures [43]. Various excitation sources can be used for active thermography, for example, laser heating, flash lamps, halogen lamps, electric heating, ultrasonic excitation, eddy currents, microwaves, and others. Inhomogeneities or defects in materials cause distortion of spatial temperature distribution and lead to temperature differences on the material surface. The main advantage of active thermography in the biomedical field is the possibility of a short thermal interaction with the sample. The thermal excitation should be shorter than the activation time of the biofeedback processes which can affect the measurement results [58].

##### Thermal Excitation: Cooling vs. Heating

Thermal agitation can be achieved by heating or cooling the sample through various approaches. The first cooling setups for active thermography of human skin are based on stimulation of the skin by conductive heat transfer using cold gel packs or balloons filled with a cold alcohol/water dispersion [60]. Large, uniformly distributed temperature gradients can be generated on the skin lesion, but some difficulties arise. These cooling methods use conductive heat transfer which means that the cooling devices touch the skin lesion. It is therefore almost impossible to monitor the surface temperature of the tissue during the thermal excitation, and accurate synchronization between the IR detector and the thermal excitation setup is difficult to achieve. Conductive cooling limits the acquisition of quantitative information [13].

For active thermography, heat can also be used as thermal excitation. Human skin can be heated by absorption of electromagnetic radiation, conduction or convection. Visible light cannot be used due to the different pigmentation of the lesion compared to the surrounding skin, which would result in heterogeneous heating [13]. A SWIR radiation source (about 2 μm) can be used to heat the skin because the absorption of the skin in IR is high and less affected by pigmentation [61]. When heating the skin, only a smaller heat gradient can be produced due to the limited heating temperature of 42 °C, a higher value would damage the living cells [58].

According to Bonmarin et al. [13], convective heat transfer is probably the optimal thermal excitation method for dermatological applications. The airflow can be regulated in temperature and relatively large temperature gradients can be exerted. Due to convective heat transfer, the skin surface temperature can be monitored with a IR camera without any obstacles. This is a noncontact thermal excitation method that contributes to a hygienic and optimal clinical device.

This led to the conclusion that cooling, although technically more difficult, is the better solution. Cooling the skin to 4 °C is acceptable, resulting in greater thermal contrasts. The large temperature gradient can be accurately measured and makes it possible to show the dominant internal heat flows, which resemble the internal structure of a tested region [58].

#### 2.2.3. Lock-In Thermography

**Figure 3:**
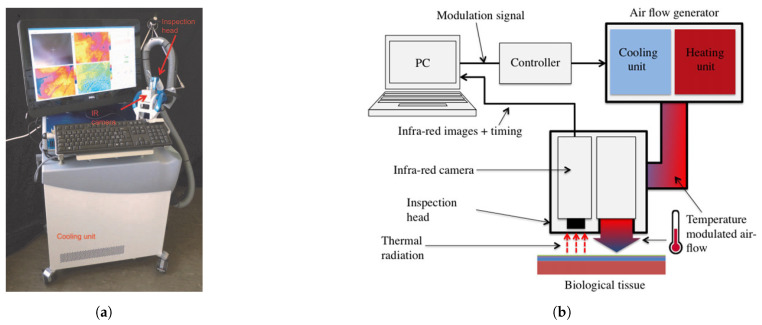
Lock-in (**a**) Lock-in device. Reprinted with permission from Ref. [62]. 2014, John Wiley & Sons A/S; (**b**) Description of lock-in setup. Reprinted with permission from Ref. [62]. 2014, John Wiley & Sons A/S.

Lock-in thermography or thermal wave imaging is commonly used in industrial environments where nondestructive testing of materials is required [62]. Heat is periodically introduced at a specific lock-in frequency and the local surface temperature modulation is evaluated and averaged over a number of periods [63]. The resulting surface temperature oscillations allows the detection of variations in the thermophysical properties under the surface of the sample [64]. Compared to steady state thermography, lock-in thermography has a higher signal to noise ratio (SNR) [63]. The phase signal with varying lock-in frequencies can reveal anomalies at different depths [63]. The high SNR has the advantage that it allows us to amplify the defect signals to make it more visible in the phase image. The in-phase and out-of-phase signals can be calculated using Equations (2) and (3), respectively [63]. Bonmarin et al. [62] presented a lock-in thermal imaging setup for a proof of concept study on benign lesions as shown in Figure 3a,b. Bhowmik et al. [65] conducted a numerical study for the detection of subsurface skin lesions using frequency modulated thermal wave imaging (FMTWI). FMTWI is an improved technique that is faster than lock-in thermography, and provides better resolution of deeper defects with a lower peak power incident heat flux [65].

(2) $$S_{0}\left(i,j\right)=\sum_{t=0}^{T}\left(\sin\left(2\pi ft\right)*F(i,j,t)\right)$$

(3) $$S_{90}\left(i,j\right)=\sum_{t=0}^{T}\left(-\cos\left(2\pi ft\right)*F(i,j,t)\right)$$

with:

$$\begin{array}{cc} f & =\mathrm{Lock-in}\;\mathrm{frequency}\\ F(t) & =\mathrm{Intensity}\;\mathrm{of}\;\mathrm{the}\;\mathrm{pixel}\;\left(i,j\right)\;\mathrm{at}\;\mathrm{time}\;t\\ T & =\mathrm{Total}\;\mathrm{time}\;\mathrm{per}\;\mathrm{measurement} \end{array}$$

The amplitude *A* and phase ϕ can be calculated with Equations (4) and (5):

(4) $$A=\sqrt{S_{0}^{2}+S_{-90}^{2}}$$

(5) $$\phi =\arctan\left(\frac{-S_{-90}}{S_{0}}\right)$$

### 2.3. Infrared Cameras

This section is intended to provide a brief overview of the most important aspects in the selection of IR cameras for dermatological applications. A detailed look at the state-of-the-art IR camera technology is beyond the scope of this review but can be found in the numerous reviews on this topic [66,67,68,69]. For a comparison of IR detector technologies chosen by other research groups in their studies, see Section 4.3. The most important minimum specifications for clinical IRT devices, according to the International Academy of Clinical Thermology (IACT) thermography guidelines [70] are listed here:

- Spectral response of 5–15 μm with a peak around 8–10 μm.
- NETD of <80 mK
- Minimal accuracy of +/−2%.
- Spatial resolution of 1 mm${}^{2}$ at a measuring distance of 40 cm from the detector.
- Fast real-time capturing of infrared data
- Absolute resolution: >19,200 temperature points
- Instantanious Field of View: <2.5 mRad
- Emissivity ϵ set to 0.98 (human skin)

#### 2.3.1. Spectral Range

**Figure 4:**
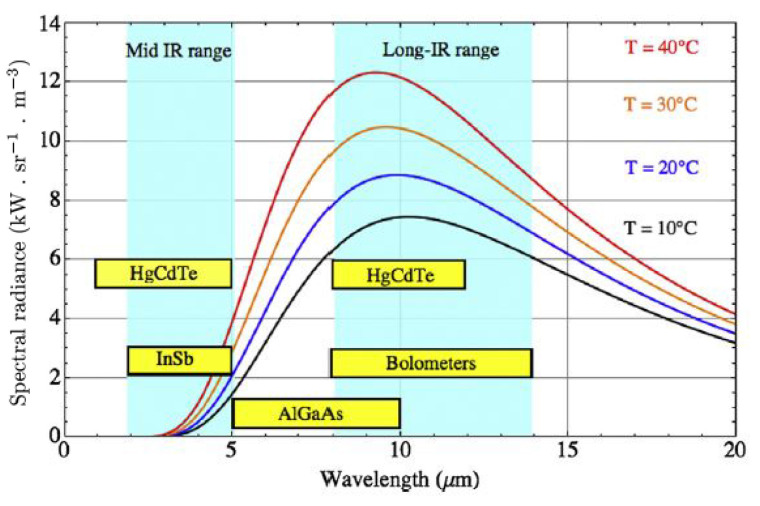
The spectral range of different IR detectors in relation to the spectral radiance of a blackbody at different equilibrium temperatures. Reprinted with permission from Ref. [13]. 2016, Elsevier.

The spectral range is the range of wavelengths that the sensor in the camera can detect. The range of different IR detectors is shown in Figure 4. Body infrared rays are in the narrow wavelength range of 8–12 μm peaking around 10 μm. It is obvious that infrared cameras in the long wave infrared range are most suitable for this application.

#### 2.3.2. Noise-Equivalent Temperature Difference (NETD)

NETD or thermal contrast is a measure of the sensitivity of IR imaging systems. NETD is a measure of the ability to distinguish between small differences in thermal radiation in the image and is expressed in milli-Kelvin at a given object temperature [68]. The lower the NETD (and thus the noise), the smaller the temperature differences that can be measured.

#### 2.3.3. Emissivity

The emissivity value is an important setting in IR cameras and therefore must be adjusted in the camera settings. The measured surface temperature is a function of emissivity, so the emissivity must be well defined [36]. Reflected temperature has less effect on measurements of a higher emissivity object and can be eliminated by choosing an appropriate value for the emissivity. For objects with high emissivity, the emissivity and reflected temperature (temperature of the environment) should be adjusted in the camera settings to automatically compensate for the influences [23]. Human skin has a high emissivity (0.98±0.01 for λ>2μm), as explained in Section 1.2. If the emissivity of the IR camera is set to ϵ=0.98 and the skin has a lower emissivity, the temperature displayed by the infrared camera will be lower than in reality. Otherwise, if the emissivity of the skin is higher, e.g., ϵ=0.98 or ϵ=0.99, the temperature displayed by the IR camera will be higher than in reality. This explains the importance of setting the emissivity correctly in the camera settings. If measurements are made from low emissivity objects, the temperature values will be inaccurate due to the high reflectance of the object. Only if the emissivity of an object is known, an IR camera can compensate the emissivity and calculate the true temperature. The radiation of the atmosphere can be neglected due to the short measurement distance and the high transmittance of the air [36].

## 3. State-of-the-Art Research: Overview

Table A1 lists the most comprehensive publications focusing on the measurement setup, procedures, and equipment for skin cancer detection with infrared thermography. Due to technological advances in the last two decades, only studies from the last twenty years are included, although the use of IR thermography to study malignant melanoma was described by other research groups in the previous century [60,71,72,73,74,75]. Currently, there is no standardized, reliable, quantitative, and noninvasive method based on IRT to accurately determine the malignant potential of skin lesions [12]. Godoy et al. [76] presented a standardized analysis protocol to analyze subject data with high sensitivity and high specificity. Their focus was on standardizing the analysis of the data, but standardization of the cooling protocol and measurement procedure is not discussed.

Buzug et al. conducted [27,77] a proof-of-concept study in 2006 in which active thermography-based assessment of skin lesions showed promise. They conducted a small clinical trial on two lesions, a basal cell carcinoma and a dysplastic nevus. In 2009, Santa Cruz et al. [78] investigated the suitability of DIRT imaging for follow-up of patients with nodular melanoma treated with boron neutron capture therapy (BNCT). Çetingül et al. [12,79,80,81,82,83,84,85,86] developed a transient thermal imaging system to accurately measure temperature differences on the skin surface to aid in the detection and diagnosis of metabolically active or malignant skin lesions. Cheng et al. [87,88,89,90,91] optimized the measurement method, skin cooling, and motion tracking of the thermal imaging system developed by Çetingül. Godoy et al. [9,76] proposed a standardized detection theory using dynamic thermography to detect the most common types of skin cancer. Inostroza [92] and Diaz [93] presented an instrument based on multimodal image registration that implements an automated procedure for early detection of skin cancer using dynamic thermal imaging. Magalhaes et al. [94,95,96,97] used different machine learning classifiers for static and dynamic thermal images of skin lesions. They did not investigate different cooling or acquisition methods. In contrast to the other research mentioned earlier, Flores-Sahagun et al. [40], Shada et al. [98] and Stringasci et al. [99,100] used passive thermography to distinguish and differentiate malignant from benign lesions.

## 4. IR Thermography in Skin Cancer Research

### 4.1. Measurement Procedure

Preparation of the patient is an important part that cannot be easily avoided. Patient acclimatization should be conducted in a room with controlled temperature and humidity. The measurement procedure should follow a clear protocol that ensures reproducibility of thermal imaging.

#### 4.1.1. Patient Preparation, Acclimatization and Controlled Environment

A standardized protocol for thermal image acquisition was developed to allow comparison of thermographic data obtained at different locations/times. The draft of this standardized protocol was presented at the IEEE EMBS conference in Amsterdam, the Netherlands, in 1996 [42,59]. The most important requirement for clinical applications of thermal imaging is a temperature-controlled environment. The temperature of the examination room should be stable and in the range of 18 to 23 °C [59]. Stability of room temperature is critical due to the human thermoregulatory system. Temperatures below 18 °C will cause shivering, while temperatures above 23 °C will cause sweating (depending on the climate) [70,101]. Air conditioners should be placed so that no airflow is directed at the patient [59].

Patient preparation is also important to obtain high quality data. The patient should remove appropriate clothing and jewelry if necessary. Acclimatization time is at least 10 min to achieve stable blood pressure and skin temperature [102,103]. Drug treatments, tight-fitting clothing, previous physical activity, and alcohol consumption may affect the skin temperature [59,104].

#### 4.1.2. Imaging Procedure

See Table A2, for an overview of the image acquisition methods used by the various research groups considered in this review. A reproducible measurement protocol must be established so that the thermal excitation of the skin is the same in all possible situations [77].

### 4.2. Measurement Setup

A measurement setup designed to capture passive or active thermography data consists out of a thermal excitation setup, camera positioning system, camera and a data acquisition system.

#### 4.2.1. Skin Excitation

If the skin lesion is examined dynamically, thermal provocation must be performed. Heating of the skin is limited to 42 °C due to the denaturation process of proteins [58]. Cooling the skin, taking into account the patient’s physical limitations can produce significant temperature differences in a short time to enhance the thermal contrast between the lesion and the healthy skin. To analyze the cooling method, different computational skin models [84,91,105,106,107,108] based on Pennes’ bioheat equation [109] are proposed.

Heat transfer in soft, living tissue can be described by Pennes’ bioheat equation. The total energy exchange through the flowing blood is proportional to the volumetric heat flux and the temperature difference between the blood and the tissue [110]. The three-dimensional expression of Pennes’ bioheat equation for soft tissue with uniform material properties is given by [109]:

(6) $$\rho C\frac{dT}{dt}=k\frac{d^{2}T}{dx^{2}}+k\frac{d^{2}T}{dy^{2}}+k\frac{d^{2}T}{dz^{2}}+\omega_{b}C_{b}\left(T_{a}-T\right)+Q_{m}+Q_{r}\left(x,y,z,t\right)$$

where:

*T* = Temperature [°C]
ρ = Tissue density [kg/m${}^{3}$]
*C* = Tissue specific heat [J/(kg °C)]
*k* = Tissue thermal conductivity [W(m °C)]
$\omega_{b}$ = Blood perfusion rate [kg/(m${}^{3}$s)]
$C_{b}$ = Blood specific heat [J/(kg °C)]
$T_{a}$ = Arterial temperature [°C]
$Q_{m}$ = Metabolic heat generation rate [W/m${}^{3}$]
$Q_{r}$ = Regional heat sources [W/m${}^{3}$]

A skin model provides information on the depth of cooling required to produce maximum thermal contrast related to cooling method, cooling temperature, and cooling time while reducing patient discomfort. Cheng et al. [91] identified two types of thermal responses. Short cooling produces maximum thermal contrast within the first few seconds of thermal recovery. Longer cooling produces maximal contrast at a later time point, i.e., 20–40 s after cooling is removed. Discussion of skin models is outside the scope of this review.

Cooling methods used by various research groups are summarized in this section. Buzug et al. [27] use a direct contact cooling method with cooled gel packs. An area of 10 cm by 10 cm is cooled to 20 °C. Santa Cruz et al. [78] use immersion in water at 15 °C for 2 min. When immersion in water was not possible, forced evaporation with an alcohol spray and fan currents was used. Santa Cruz et al. [78] concluded that a noncontact system using cold air currents was necessary to collect qualitative data without damaging the skin or causing changes in skin permeability. Cetingül et al. [82] introduced cooling with a stream of cold air from an Exair vortex tube for a period of one minute. Compressed air must be present for the vortex tube to operate. An area with a diameter of 5 cm is cooled by the outlet of the Exair vortex tube, but the cooling temperature is not mentioned in their work. Godoy et al. [9,76] proposed a standardized analysis protocol for active thermography that compensates for deficiencies in the cooling process. Godoy et al. continued to work on the research of Cetingül et al. and used a Ranque–Hilsch vortex tube as the initial cooling unit. They later replaced the cooling unit with a commercial air-conditioner because it is portable and provides a constant flow of conditioned air. In their work, no cooling temperature is specified for the conditioned air flow. Magalhaes et al. [97] used an aluminum medallion with a diameter and height of 50 mm and 20 mm, respectively, for thermal provocation of 1 min; the temperature of the cold provocation is not mentioned. Gomboc et al. [111] modeled and designed a constant temperature cooling device for melanoma screening. The device is based on an active cooling device that uses a Peltier module and a metal disk to achieve a constant cooling temperature by conduction and to induce deep cooling.

Flores-Sahagun et al. [40], Shada et al. [98] and Stringasci et al. [99,100] use passive thermography to differentiate between benign and malignant skin. Passive thermography does not require skin stimulation.

#### 4.2.2. Camera Positioning System

**Figure 5:**
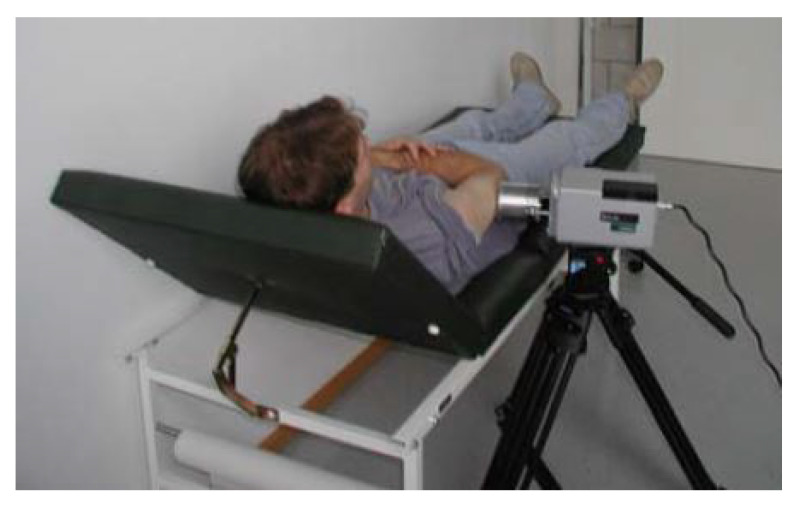
IR camera with macro lens placed on a tripod. Reprinted with permission from Ref. [27]. 2006, ACTA Press.

Buzug et al. [27] use a tripod to mount the thermal imaging camera as shown in Figure 5. A macro lens is used, which means that the camera must be placed directly in front of the lesion. Because of the use of a macro lens, motion correction of the patient is essential. Santa Cruz et al. [78] use a portable IR camera to record the thermograms of the skin lesions. The patients are located at a distance of 1.5 and 3 m. The Merlin mid-wave camera used by Cetingül et al. [82] is mounted on a tripod 30cm from the patient, giving a field of view of approximately 11.7 cm × 8.4 cm with a 22 × 16 degree FOV camera lens. Cetingül et al. states that a much better resolution can be achieved by using macro lenses. Flores-Sahagun et al. [40] used a handheld camera placed at a horizontal distance of 1 m from the region of interest. This ensured the same field of view for all measurements. Godoy et al. [9,76] placed the camera on a tripod, but no extra information is provided about the distance or angle to the lesion. Stringasci et al. [99,100] used a handheld IR camera device positioned at a distance of 15 cm from the lesion (minimum focal distance). Magalhaes et al. [97] used a handheld camera at an unspecified position relative to the skin lesion. In order to generate a qualitative and unambiguous data set, it is important to mention that the thermographic images are taken at the same distance and angle.

#### 4.2.3. ROI Markers

**Figure 6:**
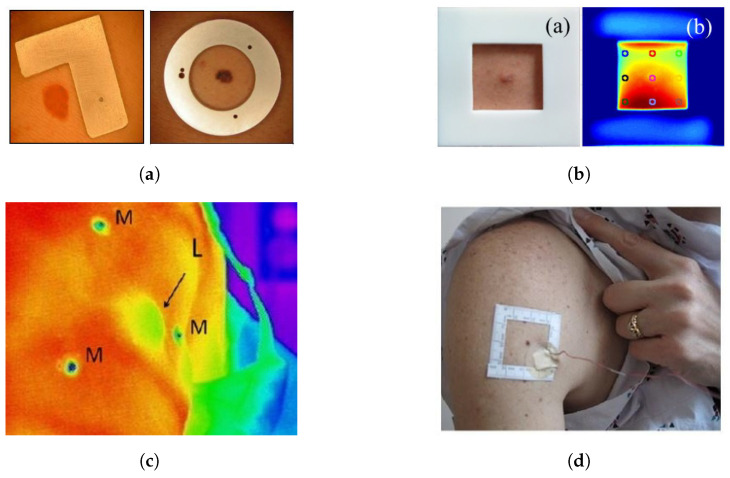
Region of interest markers for IR thermography on skin lesions used in literature. (**a**) Type of markers used by Buzug et al. Reprinted with permission from Ref. [27]. 2006, ACTA Press. (**b**) Square marker by Godoy et al. Adapted with permission from Ref. [9]. 2017, Optical Society of America. (**c**) Triangulated markers by Shada et al. Adapted with permission from Ref. [98]. 2013, Elsevier. (**d**) Square marker by Cetingül et al. Adapted with permission from Ref. [12]. 2011, JoVE.

In the steady state or after cooling the skin lesion, the entire skin surface has the same temperature. Because of the homogeneous temperature distribution, the lesion cannot be distinguished from healthy skin. To overcome this problem, Buzug et al. [27] introduced a marker that compensates for patient movement and indicates the region of interest (ROI), see Figure 6a. The marker is visible in both the white light image and the thermographic images. Patient movement during data acquisition with a macroscopic lens can lead to errors in correlation thermographic images. According to Buzug et al. [77] motion compensation is essential to automatically compare the temperatures of skin lesions and healthy skin. Santa Cruz et al. [78] immobilize the region of interest and use anatomical landmarks for image registration. Godoy et al. [9,76] used a square plastic marker with a square hole to spatially align the visible image with respect to the IR sequence (Figure 6b). Shada et al. [98] placed hypothermic markers around each lesion in a triangulating pattern to coregister the visual light and IR images (Figure 6c). Cetingül et al. [82] mark the skin lesion with a square adhesive marker centered around the lesion (Figure 6d). Stringasci et al. [99,100] applied a sticker with a millimeter scale to the skin, near the lesion. In contrast to the aforementioned studies, Magalhaes et al. [94,97] do not use an ROI marker to distinguish the region of the lesion from the surrounding benign skin.

### 4.3. Camera and Calibration

#### 4.3.1. Cameras Used in Literature

An overview of the different infrared camera systems used to study skin lesions can be found in Table A3.

#### 4.3.2. Calibration

One of the main problems of IR imaging is the indirect measurement of the sample [112]. Emissivity, sample distance, ambient temperature… affect the measured radiation. Blackbody calibration and correction of IR camera artifacts are required for quantitative measurements. IR camera nonuniformity and spatial noise should be characterized before converting the camera output to temperature [80].

Santa Cruz et al. [78] used a double-cavity black body for temperature calibration. One cavity is electrically heated to a temperature of about 40 °C and the other cavity is in equilibrium with the ambient temperature. Cetingül et al. [80] applied blackbody calibration and image degradation correction (depending on pixel position and blackbody temperature) to the infrared images. Godoy et al. [9,76] used the nonuniformity correction (NUC) tables produced by a two-point calibration at reference temperatures (25 °C and 40 °C). Magalhaes et al. [97] calibrated their camera with a blackbody ISOTECH HYPERION R982, but no calibration scheme was mentioned.

Buzug et al. [77], Flores-Sahagun et al. [40], Shada et al. [98], Stringasci et al. [99,100], Inostroza et al. [92] and Diaz et al. [93] did not report calibration details or did not calibrate the IR camera.

#### 4.3.3. Influence of Viewing Angle on Emissivity

Cheng et al. [88] quantified the curvature effect for in vivo IR thermography. The angular dependence of the surface emissivity ϵ has an effect on the measured temperature [113]. In medical diagnostics, accurate quantification of the body temperature is important to avoid biased diagnostic results.

## 5. Recommendations and Future Research

In previous research, it was often recommended to increase the number of samples included in the different datasets [12,27,62,78,94]. To improve the performance of the different detection algorithms, a larger dataset is key. The datasets should consist of different types of skin lesions (malignant and benign) [97]. Other recommendations include the use of macro lenses to achieve a higher resolution and image quality. Cooling of the skin lesion should be performed in such a way that the temperature can be monitored continuously. Convective cooling can produce large temperature gradients and is the most appropriate method for dermatologic applications [13]. Future research will focus on the developing new detection theories to distinguish of skin cancer from healthy skin [9,114]. Combining different techniques (IRT, dermoscopy, UV, ABCDE classification, numerical skin models…) to obtain more information about skin lesions may be an interesting path to explore [38,87,115].

## 6. Conclusions

Infrared thermal imaging is an inexpensive and, most importantly, noninvasive imaging technique that can be used to visualize thermographic patterns of skin lesions with relative ease. It has been used for the diagnosis of skin cancer lesions such as melanocytic nevi, basal cell carcinoma, squamous cell carcinoma, actinic keratosis and, various other diseases. Several research groups are conducting research in the field of infrared thermography on skin lesions. Preliminary results are promising, but detection of early-stage skin cancer remains difficult to achieve. Several opportunities for improvement have been identified for future work to fully exploit the potential of this technique, with a focus on dynamic thermography to improve the quality of medical diagnosis of skin cancer. The techniques used by the various researchers differ from each other. A comparative study of the different cooling methods, cameras and measurement setups on the same cohort would be interesting. The cohorts are usually small, so a large study with a measurement setup consisting of a combination of the best performing components from previous studies is needed. New techniques and cameras are constantly being introduced, so that even better results can be obtained compared to the results of previous work. Based on the research reviewed in this article, several recommendations can be made for future research.

### 6.1. Measurement Procedure

Patient preparation is important and cannot be easily avoided. A temperature-controlled environment in the range of 18 to 23 °C to perform the measurements is needed. An acclimatization period of at least 10 min is sufficient for the patient to achieve a stable blood pressure and skin temperature. A reproducible measurement protocol must be established so that the thermal excitation is the same in all possible situations.

### 6.2. Measurement Setup

The positioning of the camera affects the accuracy of the measurements. The camera should be at the same distance and angle to be able to generate a qualitative and unambiguous data set. Patient motion correction is of great importance to be able to follow the thermal recovery of the skin lesion. This is especially important when using a macro lens which provides very high-resolution images at close range but with a small field of view and focal depth. An ROI marker can be used to compensate for patient movement and distinguish the skin lesion from healthy skin.

### 6.3. Thermal Excitation

Cooling of the skin lesion is preferred because of the high thermal contrast that it can achieve. Several conclusions should be noted for the application of thermal stimulation to the skin.

- Biological tissue should not be heated to more than 42 °C while cooling of skin tissue is limited to 4 °C.
- Uniform thermal excitation is important to achieve a high degree of accuracy and high thermal contrast. Uneven cooling will result in differential thermal recovery of the skin lesion and surrounding healthy skin.
- Noncontact skin excitation such as convective cooling or heating is preferred in daily medical diagnostic practice. The skin excitation can be monitored for the physical limits of the patient with the thermal camera. Aseptic conditions can be easily ensured.

### 6.4. Camera

Quantitative measurements require calibration and correction for IR camera artifacts. Correction for nonuniformity and spatial noise should be characterized before converting the camera output to temperature. The infrared emissions from human skin at 27 °C are referred to as body infrared rays, a narrow wavelength range of 8–12 μm. An LWIR thermal imaging camera with a spectral range of about 7–14 μm is best suited for this application. Higher resolution can be achieved by using a macro lens.

## Data Availability

Not applicable.

## Abbreviations

The following abbreviations are used in this manuscript:

TBSE	Total Body Skin Examination
IRT	Infrared Thermography
DIEP	Deep Inferior Epigastric Perforator
IR	Infrared
NIR	Near-Infrared
MIR	Mid-Infrared
FIR	Far-Infrared
SWIR	Short-Wave Infrared
MWIR	Mid-Wave Infrared
LWIR	Long-Wave Infrared
VLWIR	Very-Long Wave Infrared
TIR	Thermal Infrared
SNR	Signal-Noise Ratio
FMTWI	Frequency Modulated Thermal Wave Imaging
IACT	International Academy of Clinical Thermology
NETD	Noise-Equivalent Temperature Difference
DIRT	Dynamic Infrared Thermography
BNCT	Boron Neutron Capture Therapy
ROI	Region Of Interest
NUC	Nonuniformity Correction

## Appendix A

**Table A1:** Overview of included research in this review manuscript.

Title	Authors	Year	Measurement Method	Analysis Scheme	Lesion types	Other Diagnosis Methods
**Skin-tumour classification with functional infrared imaging** [27]	Buzug et al.	2006	Quantitative	Active thermography	1 Basal-cell carcinoma 1 dysplastic nevus	histopathology
**Dynamic infrared imaging of cutaneous melanoma and** **normal skin in patients treated with BNCT [78]**	Santa Cruz et al.	2009	Quantitative	Active thermography	2 Malignant Melanoma	CT high resolution Doppler ultrasound
**The Assessment of Melanoma Risk Using the Dynamic Infrared Imaging Technique** [116]	Cetingül et al.	2011	Quantitative	Active thermography	37 dysplastic nevi of which 2 malignant melanoma	Bright light image dermatoscopic image Confocal microscopy
**Analysis and diagnosis of basal cell carcinoma (BCC) via infrared imaging** [40]	Flores-Sahagun et al.	2011	Quantitative	Passive thermography	7 basal cell carcinoma	Bright light image
**Infrared thermography of cutaneous melanoma metastases** [98]	Shada et al.	2013	Quantitative	Passive thermography	123 nonmelanomas 128 malignant melanomas	N/A
**A lock-in thermal imaging setup for dermatological applications** [62]	Bonmarin et al.	2015	Quantitative	Lock-in thermography	2 benign lesions	RGB image
**Dynamic infrared imaging for skin cancer screening** [76]	Godoy et al.	2015	Quantitative	Active thermography	59 benign lesions 29 basal-cell carcinoma 8 squamous cell carcinoma 6 malignant melanoma	RGB image
**Discrimination of benign-versus-malignant skin lesions by thermographic** **images using support vector machine classifier [100]**	Stringasci et al.	2018	Quantitative	Passive thermography	100 Basal-cell carcinoma 100 Normochromic intradermal nevus 35 Squamous cell carcinoma 35 Actinic keratosis 20 Pigmented Seborrheic Keratosis 20 malignant melanoma	RGB image
**Skin neoplasms dynamic thermal assessment** [96]	Magalhaes et al.	2019	Quantitative	Active thermography	51 Squamous cell carcinoma 118 basal cell carcinoma 16 malignant melanomas 29 actinic keratosis 30 nevi 14 seborrheic keratosis	N/A

**Table A2:** Acquisition protocols used in literature.

Author	Passive/Active Thermography	Acclimatization Time	Controlled Environment	Steady State Imaging	Cooling Type	Cooling Device	Cooling Temperature	Cooling Area	Cooling Time	Rewarming Time [s]	Number of Rewarming Frames	Camera Distance to Lesion
**Buzug et al.** **[27,77]**	Active	/	/	/	Direct contact (conduction)	Cooled gel packs	20 °C	10 cm × 10 cm	/	300	300	Directly in front
**Santa Cruz et al.** **[78]**	Active	15–20 min	/	30 s	1. Convection 2. Forced evaporation	1. immersion in water 2. alcohol spray and fan	1. 15 °C 2. /	/	1. 120 s 2. /	180	/	1.5 m and 3 m
**Cetingül et al.** **[12,83,86]**	Active	/	22 °C	1 image	Convection	vortex tube	/	/	60 s	180–240	90–120	30 cm
**Flores-Sahagun et al.** **[40]**	Passive	/	/	/	N/A	N/A	N/A	N/A	N/A	N/A	N/A	1 m
**Shada et al.** **[98]**	Passive	/	/	± 14 min	N/A	N/A	N/A	N/A	N/A	N/A	N/A	/
**Godoy et al.** **[9,76]**	Active	/	20–22 °C	15 s	1. Convection 2. Convection	1. vortex tube 2. Airconditioning unit	/		15–110 s	120 s	7200	/
**Stringasci et al.** **[99,100]**	Passive	10 min	22 °C	1 image	N/A	N/A	N/A	N/A	N/A	N/A	N/A	15 cm
**Magalhaes et al.** **[94,97]**	Active	10 min	21 ± °C relative humidity ≤50%	1 image	Direct contact (conduction)	Aluminum medal 50 mm H 20 mm	/	50 mm	60 s	300	5	/

**Table A3:** Infrared Cameras used in previous research.

Author	Buzug et al. [27,77]	Santa Cruz et al. [78]	Cetingül et al. [12,83,86]	Flores-Sahagun et al. [40]	Shada et al. [98]	Godoy et al. [9,76]	Inostroza et al. [92]	Stringasci et al. [99,100]	Diaz et al. [93]	Magalhaes et al. [94,97]
**Camera brand/type**	FLIR SC3000	Raytheon Palm IR 250	Merlin Midwave	SAT-S160	Raytheon Amber Radiance 1-T	/	FLIR Tau 2	Fluke FLK-Ti400	ThermApp	FLIR E60sc
**Detector technology**	QWIP FPA	Uncooled Ferroelectric detector FPA	InSb FPA	Uncooled Microbolometer FPA	InSb FPA	QWIP FPA	Uncooled Microbolometer FPA	Uncooled Microbolometer FPA	Uncooled Microbolometer FPA	Uncooled Microbolometer FPA
**Resolution** **[pixels]**	320 × 240	320 × 240	320 × 256	160 × 120	256 × 256	320 × 256	640 × 512	320 × 240	384 × 288	320 × 240
**Spectral Band** **[μm]**	8–9	7–14	3–5	8–14	3–5	8–14	7.5–13.5	7.5–14	7.5–14	7.5–13
**Spectral Region**	LWIR	LWIR	MWIR	LWIR	MWIR	LWIR	LWIR	LWIR	LWIR	LWIR
**Accuracy**	±1%	/	±2 %	±2%	/	/	/	±2%	±2%	±2%
**NETD**	20 mK at 30 °C	/	25 mK at 30 °C	100 mK at 30 °C	/	20 mK at 30 °C	60 mK at 30 °C	50 mK at 30 °C	70 mK at 30 °C	<50 mK at 30 °C
**Framerate** **[Hz]**	50/60	30	60	50/60	/	60	30	60	25	/
**Camera Objective**	Macro lens	75 mm Germanium lens	/	/	/	50 mm, f/2	/	/	/	/
**Fixed/Handheld**	Fixed	Handheld	Fixed	Handheld	Fixed	Fixed	Fixed	Handheld	Handheld	Handheld
**Calibration Method**	/	Double-cavity Black body	Black body calibration image degradation correction	/	/	two-point NUC	/	/	/	Black body calibration no method described
